# Fungal Community Assembly in the Amazonian Dark Earth

**DOI:** 10.1007/s00248-015-0703-7

**Published:** 2015-11-19

**Authors:** Adriano Reis Lucheta, Fabiana de Souza Cannavan, Luiz Fernando Wurdig Roesch, Siu Mui Tsai, Eiko Eurya Kuramae

**Affiliations:** Department of Microbial Ecology, Netherlands Institute of Ecology (NIOO/KNAW), Droevendaalsesteeg 10, Wageningen, 6708 PB The Netherlands; Centro de Energia Nuclear na Agricultura (CENA), Universidade de São Paulo (USP), Piracicaba, Brazil; Centro Interdisciplinar de Pesquisas em Biotecnologia (CIP-Biotec), Universidade Federal do Pampa, São Gabriel, Brazil

**Keywords:** 18S rRNA, Anthrosols, Biochar, Microbial ecology, Pre-Columbian soil, Pyrosequencing

## Abstract

Here, we compare the fungal community composition and diversity in Amazonian Dark Earth (ADE) and the respective non-anthropogenic origin adjacent (ADJ) soils from four different sites in Brazilian Central Amazon using pyrosequencing of 18S ribosomal RNA (rRNA) gene. Fungal community composition in ADE soils were more similar to each other than their ADJ soils, except for only one site. Phosphorus and aluminum saturation were the main soil chemical factors contributing to ADE and ADJ fungal community dissimilarities. Differences in fungal richness were not observed between ADE and ADJ soil pairs regarding to the most sites. In general, the most dominant subphyla present in the soils were *Pezizomycotina*, *Agaricomycotina*, and *Mortierellomycotina*. The most abundant operational taxonomic units (OTUs) in ADE showed similarities with the entomopathogenic fungus *Cordyceps confragosa* and the saprobes *Fomitopsis pinicola*, *Acremonium vitellinum*, and *Mortierellaceae* sp., whereas OTUs similar to *Aspergillus niger*, *Lithothelium septemseptatum*, *Heliocephala gracillis*, and *Pestalosphaeria* sp. were more abundant in ADJ soils. Differences in fungal community composition were associated to soil chemical factors in ADE (P, Ca, Zn, Mg, organic matter, sum of bases, and base saturation) and ADJ (Al, potential acidity, Al saturation, B, and Fe) soils. These results contribute to a deeper view of the fungi communities in ADE and open new perspectives for entomopathogenic fungi studies.

**Electronic supplementary material:**

The online version of this article (doi:10.1007/s00248-015-0703-7) contains supplementary material, which is available to authorized users.

## Introduction

Amazonian Dark Earth (ADE), also referred to as “*Terra Preta*”, was described by Sombroek [1] as a “well-drained soil characterized by the presence of a thick black or dark gray topsoil which contains pieces of artifacts”. The anthropogenic, pre-Columbian soils occur in 20-ha average spots in the Amazonian region [2]. ADE is recognized by the elevated amounts of stable carbon (70 times more black carbon) and fertility due to the high concentration of P, Ca, Mg, and Zn, nutrient holding capacity, and higher pH when compared to adjacent non-anthropogenic origin soils [3, 4]. Despite evidences of human occupation in the Amazon region dating 10,000 years BP (before present), Neves and co-workers [5] suggested that ADE formation occurred 2500 to 2000 years ago as a result of population increasing during that period. It is still unclear if the ADE was intentionally created or if it was a result of disposals by native settlements. However, the consensus is that the main sources of ADE nutrients originated from human and animal excrements, plant and animal residues, mammalian and fish bones, housing material and pottery debris, ash, and charred organic materials [2, 5, 6].

Another remarkable characteristic of ADE is the elevated microbial diversity and associated bacterial species richness [7]. Using culture dependent and independent approaches, studies reveal bacterial and archaeal communities in ADE that are distinct from the adjacent soil or from isolated black carbon [8–10].

Significant advances in soil microbial ecology studies were obtained in the last few years after the adoption of high-throughput 16S ribosomal RNA (rRNA) gene sequencing technologies [11]. This approach was used to investigate the bacterial community associated with biochar samples of ADE [12] and the effect of ADE and plant species on the selection of rhizosphere bacterial communities [4]. However, the fungal communities associated with ADE have not yet been investigated with culture-independent methods despite the ecological importance of fungi in terrestrial ecosystems. The degradation of organic matter, mainly by saprophytic fungi, controls the balance between soil and atmospheric carbon and releases nutrients for plant uptake [13–15]. The fungal community in ADE has been poorly characterized and evaluated only by low-resolution culture-dependent methods [16]. The application of high-throughput sequencing technologies will expand the knowledge of ADE fungal diversity. Comparison to low fertility adjacent soils will help to elucidate the carbon transformations by fungi in these soils and to evaluate potential land use and climate change effects for future studies. Therefore, the aim of this study was to estimate the fungal richness and diversity associated to ADE and to adjacent soils from four sites in the Central Amazon through pyrosequencing of 18S rRNA gene fragments.

## Materials and Methods

### Site Description and Soil Sampling

The study area was comprised of four locations in the Brazilian Central Amazonia region near Manaus, the capital of Amazonas state (AM). ADE and the respective adjacent (ADJ) non-anthropogenic origin soils were collected at (1) Açutuba (ACU, 03° 05′ 53.92″ S, 60° 21′ 19.90″ W), located at the margin of Negro River close to the municipality of Iranduba (AM), under cultivation of eggplant (ADE) and pasture (ADJ) at the time of sampling; (2) Balbina (BAL, 01° 30′ 24.4″ S, 60° 05′ 34″ W), located at the Presidente Figueiredo municipality and characterized by the presence of an undisturbed secondary forest. This site has not been deforested or used for agriculture purposes for at least 20 years [17]; (3) Hatahara (HAT, 03° 16′ 28.45″ S, 60° 12′ 17.14″ W) located in a bluff on the margin of Solimões river cultivated with banana plants (ADE) and pasture (ADJ). This is one of the most studied archaeological ADE sites in Central Amazon [18]; (4) Barro Branco (BBO, 03° 18′ 24.76″ S, 60° 32′ 5.10″ W), located upstream Hatahara in the margin of Solimões River close to Manacapuru (AM) under a citrus orchard (ADE) and cassava plantation (ADJ).

The soil sampling scheme in each site was set by a geo-referenced central point (A) and four extra points 1.5 m distant in the cardinal direction (B, C, D, E). Each soil sample was composed by five subsamples (A1, A2, A3, A4, A5, B1, B2, B3, B4, B5, etc.) collected 0.3 m around the main point at 0–10 cm depth using sterile plastic cylinders (5-cm diameter). Sampling scheme illustration can be viewed in Online Resource 1. To minimize the current land use effect, grass layer and litter were removed, and then, the soil samples were collected in the space between rows when cultivated. Soil samples were kept on dry ice before storage at −20 °C. Soil physicochemical attributes were determined following Raij et al. [19] in the Soil Fertility Laboratory of the Department of Soil Sciences, University of São Paulo (ESALQ-USP). Fieldwork was conducted under legal authorization (SISBIO 4845833).

### DNA Extraction, Amplification, and Sequencing of 18S rRNA Gene Fragment

Total DNA was extracted from 250 mg of bulk soil in triplicate from only three of the five soil samples (A, B, D) using the Power Soil DNA isolation kit (MO BIO Laboratories Inc., Carlsbad, CA, USA) following the manufacturer’s instructions. Extracted DNA was quantified using a NanoDrop ND-1000 spectrophotometer (Thermo Scientific, Wilmington, DE, USA). The 18S rRNA gene fragments were amplified by polymerase chain reaction (PCR) using 0.5 μM of the fungal-specific reverse primer FR1 [20] and the modified version (to include Glomeromycota arbuscular mycorrhizal fungi) of forward primer FF390w (5′-CGWTAACGAACGAGACCT-3′) [21]. Four PCR reactions (25 μL) per extracted sample DNA were carried out using 2.5× reaction buffer containing 18 mM of MgCl_2_, 0.2 mM of each dNTP, 0.5 μM of each primer, 25 ng of template DNA, 1 U Taq polymerase FastStart High Fidelity (Roche Applied Sciences, Indianapolis, IN, USA), and sterile water to 25 μL final volume. The thermocycling conditions were initial denaturing at 94 °C for 4 min, 29 cycles of 94 °C for 30 s, 55 °C for 1 min (annealing temperature was lowered 2 °C every 2 cycles until 47 °C), and extension at 68 °C for 2 min. The technical PCR replicate (12 PCR reactions/soil replicate) amplicons were pooled and cleaned with the Qiagen PCR purification kit (Qiagen, Valencia, CA, USA) to avoid amplification bias. A total of 24 soil samples (4 sites × 2 soil types × 3 replicates) were amplified using barcoded primers (MID tags) for multiplex pyrosequencing in a Roche 454 GS FLX automated sequencer (454 Life Sciences, Brandford, CT, USA) using titanium chemistry. The complete list of barcoded primers is listed in Online Resource 2.

### Bioinformatics and Statistical Analysis

The 18S rRNA gene sequences were analyzed using QIIME 1.8.0 [22] following the suggested 18S data analysis tutorial (http://qiime.org/1.8.0/tutorials/processing_18S_data.html). Multiplex sequence libraries were split into the original samples based on the specific barcodes. The 454 reads were denoised using Denoiser [23] and chimeric sequences checked with UCHIME [24]. Operational taxonomic units (OTUs) were clustered considering evolutionary distance of 0.03 (97 % similarity cutoff) by using UCLUST algorithm [25] and taxonomically affiliated through BLAST search using QIIME BLAST Taxon Assigner default parameters (application blastn/megablast, max *E* value 0.001, min percentage identity 90.0) against SILVA Eukaryotic database (97 SILVA 111 rep set euk) [26, 27]. OTUs not assigned to Fungi kingdom, singletons (OTUs containing a unique sequence in the whole analysis) as well as classified as “no hit” by BLAST search were removed from the dataset. Inconsistences of SILVA taxonomic classification were manually corrected before relative abundance calculation based on the OTU BLAST search best hit access number and NCBI taxonomy rank (http://www.ncbi.nlm.nih.gov/taxonomy). The OTU table was rarefied to the lowest number of sequences in any sample (1728) before calculation of alpha diversity indices. Species richness (Chao1 and Abundance Coverage-based Estimator (ACE)), diversity (Shannon, Simpson’s reciprocal) estimators, Good’s coverage, and rarefaction curves were calculated in QIIME. Chao entropy index [28] was calculated on the CHAOEntropy-Online calculator (https://yuanhan.shinyapps.io/ChaoEntropy/). A bipartite OTU network was generated in QIIME and viewed and edited in Cytoscape 3.2.1 [29]. The fungal OTUs present in all soil samples (total core) (core_table_100.biom file), as well as the common OTUs belonging to ADE or ADJ soils (group core) were also determined in QIIME (compute_core_microbiome.py). OTUs showing average abundance higher than 1 % of the total number of sequences by group (ADE or ADJ) were considered abundant. Differential OTU frequencies between ADE and ADJ soil groups was determined by non-parametric *t* test followed by Monte Carlo test (100 replicates) after removing the OTUs that were not represented in at least 25 % of the samples using QIIME (group_significance.py). Univariate analyses (*t* test, ANOVA, Tukey’s test) were performed using IBM SPSS Statistics V.22 (IBM Corp., Armonk, NY, USA) software, whereas multivariate analyses (canonical correspondence analysis and similarity percentage analysis) were performed using paleontological statistics (PAST) software package V.3.05 [30]. Count data (sequence abundances) and environmental variable values were transformed (function Log(*x* + 1)) before multivariate analysis. The raw 454 pyrosequencing data of the 18S rRNA are available at the European Nucleotide Archive (ENA) (https://www.ebi.ac.uk/ena/) under the study accession number PRJEB10851.

## Results

### Soil Physicochemical Properties

All the evaluated soil physicochemical and fertility attributes were statistically different (*p* ≤ 0.05) when ADE and ADJ soils were compared in groups, with the exception of the K, S, and Fe attributes. On average, ADE soils were higher in pH, organic matter (OM), macronutrients (P, Ca, Mg), and some micronutrients (Mn, Cu and Zn), while ADJ soils had higher levels of Al and H + Al (Table 1). Within the ADE soil group, the Hatahara sample showed the highest amounts of P, Cu, Fe, Zn, and Mn, whereas in the ADJ soil group, the Açutuba soil sample showed the highest amount of K and lowest Al concentration and Al saturation, comparable with ADE samples (Table 1).

**Table 1:** Physicochemical and fertility attributes of Amazonian Dark Earth (ADE) and adjacent (ADJ) soils

Properties	ADE				ADJ				ADE versus ADJ^b^
	Açutuba	Balbina	Barro Branco	Hatahara	Açutuba	Balbina	Barro Branco	Hatahara	
pH	4.87 ± 0.64ab^a^	4.73 ± 0.76abc	5.17 ± 0.15a	5.20 ± 0.2a	4.6 ± 0.4abc	3.93 ± 0.15bcd	3.47 ± 0.12d	3.73 ± 0.12cd	***
OM	21.67 ± 16.44b	49.0 ± 4.58a	54.67 ± 9.24a	53.0 ± 2.65a	36.0 ± 7.81ab	32.33 ± 10.69ab	34.67 ± 5.69ab	33.33 ± 1.15ab	*
P	204.67 ± 168.36b	90.0 ± 49.43bc	130.0 ± 34.04bc	508.67 ± 88.64a	41.0 ± 19.16bc	2.67 ± 1.53c	3.67 ± 2.08c	7.33 ± 1.15bc	**
K	1.0 ± 0.17b	0.30 ± 0.0b	1.03 ± 0.35ab	1.07 ± 0.25ab	3.13 ± 2.02a	0.70 ± 0.36b	0.43 ± 0.06b	0.67 ± 0.06b	ns
Ca	57.67 ± 50.82bc	44.67 ± 49.69bc	86.33 ± 14.19ab	138.33 ± 7.64a	35.67 ± 17.04bc	3.33 ± 2.31c	1.67 ± 0.58c	6.33 ± 1.15c	***
Mg	5.33 ± 2.31bc	2.67 ± 2.08c	10.67 ± 1.15ab	13.67 ± 3.06a	6.0 ± 3.61bc	2.00 ± 1.73c	1.0 ± 0.0c	1.0 ± 0.0c	**
S	3.33 ± 0.58b	3.67 ± 0.58b	4.33 ± 0.58b	4.33 ± 0.58b	5.0 ± 1.0b	3.67 ± 0.58b	8.33 ± 2.08a	3.67 ± 0.58b	ns
SB	64.0 ± 53.29bc	47.63 ± 51.73bc	98.03 ± 14.36ab	153.07 ± 5.39a	44.80 ± 21.02bc	6.03 ± 4.39c	3.1 ± 0.61c	8.0 ± 1.13c	***
CEC	114.00 ± 34.04b	125.63 ± 17.21b	140.37 ± 14.2ab	200.73 ± 0.6a	101.80 ± 10.69b	79.37 ± 39.2b	135.1 ± 30.29ab	103.67 ± 15.71b	*
V%	50.67 ± 27.59ab	35.0 ± 33.81abc	69.67 ± 4.04a	76.00 ± 2.65a	43.0 ± 17.35abc	9.33 ± 8.5bc	2.33 ± 0.58c	7.67 ± 0.58bc	***
B	0.32 ± 0.17ab	0.17 ± 0.05ab	0.21 ± 0.1ab	0.10 ± 0.09b	0.23 ± 0.05ab	0.37 ± 0.18ab	0.48 ± 0.09a	0.32 ± 0.11ab	*
Cu	1.2 ± 0.35b	0.63 ± 0.06bc	1.13 ± 0.15b	3.27 ± 0.67a	0.43 ± 0.23bc	0.0 ± 0.0c	0.0 ± 0.0c	0.03 ± 0.06c	***
Zn	4.3 ± 1.47bc	5.0 ± 1.11bc	7.70 ± 5.54b	30.77 ± 4.61a	1.30 ± 0.62bc	0.1 ± 0.1c	0.17 ± 0.15c	0.3 ± 0.1bc	**
Fe	48.67 ± 22.19c	36.33 ± 10.02c	66.0 ± 2.0c	210.67 ± 36.56a	70.0 ± 14.8c	90.67 ± 75.05bc	193.0 ± 47.13ab	182.33 ± 27.43ab	ns
Mn	3.6 ± 1.57bc	4.0 ± 0.69bc	4.93 ± 0.75b	12.60 ± 3.8a	1.37 ± 0.65bc	0.27 ± 0.12c	0.53 ± 0.15c	0.4 ± 0.17c	***
Al	1.0 ± 1.0d	5.0 ± 5.0 cd	0.0 ± 0.0d	0.0 ± 0.0d	2.0 ± 2.65d	13.0 ± 4.36bc	23.67 ± 3.06a	14.0 ± 1.73b	***
H + Al	50.0 ± 19.29b	78.0 ± 34.64ab	42.33 ± 4.51b	48.67 ± 5.77b	57.0 ± 13.23b	73.33 ± 41.36ab	132.00 ± 30.05a	95.67 ± 14.64ab	*
m%	3.33 ± 3.51b	20.0 ± 20.0b	0.0 ± 0.0b	0.0 ± 0.0b	7.0 ± 10.44b	69.33 ± 17.21a	88.67 ± 2.52a	63.67 ± 0.58a	***

pH (CaCl_2_ 0.01 mol L^−1^); organic matter (OM) expressed in g dm^−3^; P and S expressed in mg dm^−3^; K, Ca, Mg, sum of bases (SB), cation exchange capacity in pH 7 (CEC), Al, and H + Al expressed in mmolc dm^−3^; B, Cu, Zn, Fe, and Mn expressed in mg dm^−3^

*V* soil base saturation index (%), *m* Al saturation index (%), *ns* not significant

^a^The showed values are the average of three replicates followed by standard deviation. Same letters represent no significant differences by Tukey’s test (*p* ≤ 0.05)

^b^Independent sample *t* test comparing ADE × ADJ soil groups

*p ≤ 0.05; **p ≤ 0.005; ***p ≤ 0.0005

### Diversity of Fungal Community

#### 18S rRNA Sequencing

Pyrosequencing of 18S rRNA gene from the 24 soil samples generated 132,764 high-quality sequences after denoising and chimera checking, with an average size of 351 nucleotides. A total of 105,019 sequences were used for further analysis after taxonomic classification as fungal. The numbers of sequences per library ranged from 1728 to 6712. A detailed description of sequencing depth and number of OTUs along the bioinformatics analyses can be viewed at Online Resource 3. The number of picked OTUs ranged between 127 and 172 after the removal of singletons and library normalization with the lowest number of sequences (1728) (Table 2). Despite the decrease in the number of sequences after quality filtering and library normalization (Online Resource 3), the sample coverage was approximately 97 % as indicated by Good’s estimator (Table 2). In addition, rarefaction curves also pointed for adequate sequencing efforts for fungal population coverage in the samples (Online Resource 4).

**Table 2:** Estimated richness and diversity indices for the fungal communities in the Amazonian Dark Earth (ADE) and adjacent (ADJ) soils from Açutuba (ACU), Balbina (BAL), Barro Branco (BBO), and Hatahara (HAT) sites

		Species richness estimators		Diversity estimators			
Soil type/site	*N* OTUs^a^	ACE	Chao-1	1/D^c^	*H*′^d^	Chao entropy^e^	Good’s^f^
ADE							
ACU	136 (183, 89)^b^	200.14 (239.10, 161.17)	197.06 (245.57, 148.54)	10.30 (16.66, 3.94)	4.58 (5.86, 3.31)	3.24 (4.13, 2.35)	0.97 (0.97, 0.96)
BAL	132 (145, 119)	200.28 (251.44, 149.12)	193.01 (262.55, 123.48)	6.66 (9.27, 4.05)	4.19 (4.83, 3.56)	2.97 (3.42, 2.52)	0.97 (0.98, 0.96)
BBO	139 (177, 102)	210.90 (253.77, 168.03)	206.80 (285.52, 128.08)	7.74 (11.95, 3.53)	4.46 (5.33, 3.59)	3.16 (3.77, 2.54)	0.97 (0.98, 0.96)
HAT	172 (182, 162)	245.40 (254.92, 235.87)	248.17 (294.53, 201.81)	20.21 (30.11, 10.32)	5.60 (5.74, 5.46)	3.96 (4.06, 3.86)	0.96 (0.97, 0.96)
ADJ							
ACU	164 (173, 155)	236.48 (284.49, 188.46)	238.93 (348.00, 129.86)	13.91 (15.00, 12.82)	5.18 (5.29, 5.06)	3.66 (3.74, 3.59)	0.96 (0.98, 0.95)
BAL	166 (186, 146)	243.53 (269.82, 217.23)	246.05 (326.08, 166.02)	15.54 (19.64, 11.44)	5.22 (5.72, 4.73)	3.70 (4.03, 3.37)	0.96 (0.97, 0.95)
BBO	127 (145, 109)	191.69 (250.32, 133.07)	180.82 (221.30, 140.33)	14.95 (16.42, 13.48)	4.94 (5.10, 4.79)	3.49 (3.60, 3.37)	0.97 (0.98, 0.96)
HAT	138 (166, 110)	188.47 (231.82, 145.11)	184.10 (202.18, 166.02)	15.98 (26.94, 5.01)	5.12 (5.93, 4.30)	3.61 (4.17, 3.04)	0.97 (0.98, 0.97)

^a^Number of determined operational taxonomic units

^b^The showed values represent the average of three replicates followed by confidence intervals

^c^Simpson’s reciprocal (1/D) index

^d^Shannon index

^e^Chao entropy index (Chao et al. 2013)

^f^Good’s estimated sample coverage

No significant differences in the estimated species richness was observed by ACE and Chao1 estimators when comparing the ADE and ADJ soil samples in the same sites, with exception of a higher number of species in the Hatahara ADE sample in relation to its ADJ soil (ACE estimator). Regarding the fungal species diversity, Shannon and Chao entropy estimators also pointed to no differences between the ADE and ADJ soils. Simpson’s reciprocal indicated lower species diversity in the ADE from BAL and BBO in comparison with the respective ADJ samples (Table 2).

#### Statistical Multivariate Analysis

**Fig. 1:**
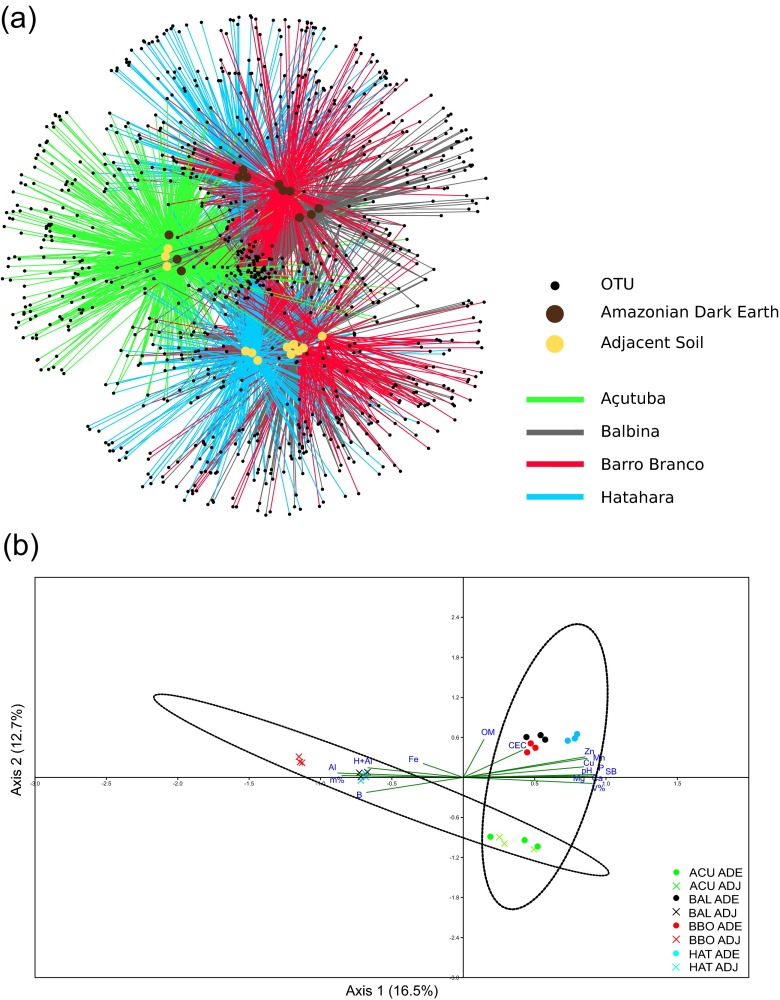
Bipartite network connecting the fungal OTU nodes to the Amazonian Dark Earth (ADE) and adjacent (ADJ) soil nodes by Açutuba (ACU), Balbina (BAL), Barro Branco (BBO), and Hatahara (HAT) representing edges (**a**) and canonical correspondence analysis (CCA) with 95 % confidence ellipses (**b**)

Even though similarities were observed in the species richness and diversity of the ADE and ADJ soil fungal communities, the OTU network and canonical correspondence analysis (CCA) showed two well-defined clusters segregating the fungal communities of ADE and ADJ soils from BAL, BBO, and HAT (Fig. 1a, b). The same pattern could not be observed for the fungal communities of ADE and ADJ soils from ACU that were more similar to each other and distant from the other site assemblages (Fig. 1b). CCA also indicated that the ADE fungal assemblages were correlated with higher pH, macronutrients, sum of bases (SB), percentage of soil base saturation (V%), and Cu, Zn, and Mn concentrations, whereas ADJ community was correlated with Al, H + Al, aluminum saturation (m%), and B levels (Fig. 1b). P and m% contributed with more than 13 % each in the ADE versus ADJ fungal community dissimilarity as calculated by similarity percentage analysis (SIMPER) (Online Resource 5). Conversely, the OM and pH contributed 2.5 and 0.9 % to the dissimilarities, respectively.

#### Fungal Taxonomy

The phylum *Ascomycota*, specifically the subphylum *Pezizomycotina*, was the most abundant in all the soil samples with exception of BAL ADE that was dominated by *Agaricomycotina* fungi (Basidiomycota) (Table 3). *Pezizomycotina* fungi were statistically significantly (*p* ≤ 0.05) more abundant in ADJ soils, and shifts were detected especially in Balbina and Hatahara sites. Ascomycota *Taphiromycotina* (*p* ≤ 0.005) and Mitosporic Acomycota (*p* ≤ 0.05), *Chytridiomycota Insertae sedis* (*p* ≤ 0.005), Fungi *Insertae sedis Mucoromycotina* (*p* ≤ 0.05), and Glomeromycota phylum (arbuscular mycorrhizal fungi) were also more abundant in ADJ soils at statistical significant level.

**Table 3:** Relative abundance (%) of the fungi 18S rRNA gene taxa (based on BLAST best hit taxonomical classification) of Amazonian Dark Earth (ADE) and adjacent (ADJ) soils from Açutuba (ACU), Balbina (BAL), Barro Branco (BBO), and Hatahara (HAT) sites

Phylum^a^	Subphylum^a^	ACU ADE^b^	ACU ADJ	BAL ADE	BAL ADJ	BBO ADE	BBO ADJ	HAT ADE	HAT ADJ	ADE versus ADJ^c^
Ascomycota	*Pezizomycotina*	44.27 ± 6.41abc	39.68 ± 8.18abc	27.37 ± 4.70c	47.28 ± 6.41ab	49.92 ± 2.21ab	51.54 ± 6.40ab	34.22 ± 8.05bc	56.73 ± 7.99a	*
	*Saccharomycotina*	0.08 ± 0.03b	0.04 ± 0.03b	0.04 ± 0.03b	0.17 ± 0.15b	0.58 ± 0.15a	0.04 ± 0.03b	0.02 ± 0.03b	0.06 ± 0.10b	ns
	*Taphrinomycotina*	0.00 ± 0.00c	0.00 ± 0.00c	0.00 ± 0.00c	0.54 ± 0.22a	0.00 ± 0.00c	0.39 ± 0.29ab	0.02 ± 0.03bc	0.19 ± 0.09abc	**
	Mitosporic Ascomycota	0.00 ± 0.00b	0.00 ± 0.00b	0.00 ± 0.00b	10.47 ± 2.28a	0.00 ± 0.00b	0.58 ± 0.31b	0.00 ± 0.00b	2.33 ± 1.34b	*
	Unclassified Ascomycota	0.46 ± 0.10a	0.15 ± 0.12b	0.02 ± 0.03b	0.12 ± 0.06b	0.00 ± 0.00b	0.08 ± 0.09b	0.02 ± 0.03b	0.08 ± 0.07b	ns
Basidiomycota	*Agaricomycotina* (no rank)	7.66 ± 3.45b	3.70 ± 1.71b	33.29 ± 4.93a	8.85 ± 1.00b	6.35 ± 0.94b	2.57 ± 0.59b	7.77 ± 1.24b	3.80 ± 1.32b	*
	Basidiomycota environmental samples	0.15 ± 0.22b	1.10 ± 0.50ab	0.10 ± 0.03b	0.79 ± 0.58b	0.14 ± 0.19b	0.19 ± 0.07b	3.92 ± 5.29ab	6.71 ± 2.28a	ns
	*Pucciniomycotina*	0.00 ± 0.00b	0.04 ± 0.03b	1.10 ± 1.10ab	0.02 ± 0.03b	1.22 ± 1.31ab	0.06 ± 0.06b	2.28 ± 0.58a	0.64 ± 0.15ab	*
	*Ustilaginomycotina*	0.00 ± 0.00b	0.00 ± 0.00b	0.00 ± 0.00b	0.02 ± 0.03b	0.00 ± 0.00b	0.21 ± 0.09a	0.04 ± 0.07b	0.00 ± 0.00b	ns
Blastocladiomycota	Blastocladiomycota *Insertae sedis*	3.43 ± 0.83ab	8.06 ± 7.35a	0.08 ± 0.07b	0.12 ± 0.15b	0.02 ± 0.03b	0.12 ± 0.12b	0.14 ± 0.19b	0.27 ± 0.19b	ns
Chytridiomycota	Chytridiomycota *Insertae sedis*	1.04 ± 0.50c	7.02 ± 1.02a	0.75 ± 0.45c	1.81 ± 1.49c	0.87 ± 0.32c	2.66 ± 0.49bc	1.62 ± 0.21c	4.78 ± 1.83ab	**
	Chytridiomycota environmental samples	1.68 ± 0.86b	4.92 ± 1.43b	0.81 ± 0.55b	1.23 ± 0.34b	12.38 ± 3.44a	9.38 ± 0.29a	2.58 ± 1.84b	3.59 ± 0.29b	ns
Entomophthoromycota	Entomophthoromycota *insertae sedis*	0.23 ± 0.27a	0.23 ± 0.27a	0.41 ± 0.15a	0.10 ± 0.09a	0.00 ± 0.00a	0.02 ± 0.03a	0.41 ± 0.50a	0.00 ± 0.00a	ns
Entorrhizomycota	Entorrhizomycota *Insertae sedis*	0.00 ± 0.00a	0.58 ± 0.67a	0.00 ± 0.00a	0.00 ± 0.00a	0.00 ± 0.00a	0.00 ± 0.00a	0.08 ± 0.09a	0.04 ± 0.03a	ns
Fungi *incertae sedis*	*Kickxellomycotina*	0.15 ± 0.09b	0.10 ± 0.17b	0.14 ± 0.03b	0.25 ± 0.15b	0.42 ± 0.13b	0.06 ± 0.06b	1.16 ± 0.12a	0.50 ± 0.57b	ns
	*Mortierellomycotina*	16.78 ± 1.33a	9.12 ± 2.38b	7.68 ± 2.61b	10.15 ± 2.57b	7.39 ± 1.77b	4.48 ± 0.54b	18.52 ± 2.61a	4.32 ± 2.60b	*
	*Mucoromycotina*	0.85 ± 0.24bc	0.79 ± 0.32c	0.44 ± 0.09c	3.05 ± 0.79ab	1.77 ± 0.59bc	4.88 ± 1.76a	0.71 ± 0.37c	2.37 ± 0.78bc	**
	*Zoopagomycotina*	0.25 ± 0.34b	0.35 ± 0.15ab	0.98 ± 0.46a	0.17 ± 0.17b	0.35 ± 0.12ab	0.02 ± 0.03b	0.50 ± 0.15ab	0.04 ± 0.07b	*
Glomeromycota	Glomeromycota *Insertae sedis*	4.07 ± 0.44ab	4.53 ± 1.24a	0.83 ± 0.26bc	4.75 ± 2.61a	0.46 ± 0.12c	1.58 ± 0.03abc	2.28 ± 1.41abc	3.63 ± 0.72abc	*
	Unclassified Glomeromycota	0.00 ± 0.00a	0.10 ± 0.07a	0.00 ± 0.00a	0.15 ± 0.03a	0.00 ± 0.00a	0.15 ± 0.07a	0.00 ± 0.00a	0.17 ± 0.25a	**
Uncultured fungus	Environmental samples	18.89 ± 6.25ab	19.46 ± 3.13ab	25.95 ± 0.35a	9.93 ± 0.49b	18.13 ± 2.69ab	21.01 ± 7.33ab	23.73 ± 5.26a	9.51 ± 3.05b	*
Unclassified sequence		0.00 ± 0.00c	0.02 ± 0.03ab	0.02 ± 0.03ab	0.02 ± 0.03ab	0.00 ± 0.00c	0.00 ± 0.00c	0.00 ± 0.00c	0.23 ± 0.21a	ns

*ns* not significant

^a^Taxonomic classification based on the NCBI rank of the best hit after BLAST search against 97 SILVA 111 database

^b^The showed values are the average of three replicates followed by standard deviation. Same letters represent no significant differences by Tukey’s test (*p* ≤ 0.05)

^c^Independent sample *t* test comparing ADE × ADJ soil groups

*p ≤ 0.05; **p ≤ 0.005

The ADE soils showed significant higher abundance of Basidiomycota *Agaricomycotina* (*p* ≤ 0.05) and *Pucciniomycotina* (*p* ≤ 0.05), Fungi *Insertae sedis Zoopagomycotina* (*p* ≤ 0.05), and *Mortierellomycotina* (*p* ≤ 0.05). Shifts in *Mortierellomycotina* abundance were observed mainly in ACU and HAT sites.

A significant number of sequences, especially in ADE (*p* ≤ 0.05), were taxonomically classified only at Fungi domain and environmental sample category based on BLAST access taxonomy rank. We cannot affirm whether these results could represent new fungal species or are resultant of SILVA and NCBI database annotation imprecision.

#### Fungal Core Community

The fungal core community present in all soil samples and locations computed in QIIME was composed of seven OTUs, most of them classified as Ascomycota phylum (Table 4). At species level, they showed similarity to Ascomycota *Cordyceps confragosa* (OTU 822), *Lithothelium septemseptatum* (OTU 1344), *Aspergillus niger* (OTU 2196), *Ophiocordyceps clavata* (OTU 2207), and *Fomitopsis pinicola* (Basidiomycota, OTU 153), and OTUs 874 and 1924 were classified as uncultured fungus (Table 4).

**Table 4:** Amazonian Dark Earth (ADE) and adjacent (ADJ) soils general fungal OTU core and specific soil type cores (ADE or ADJ) followed by the best BLAST hit and the NCBI taxonomical classification

			NCBI taxonomic classification^a^		
OTU number	Soil group	Access number	Phylum	Subphylum	Specie
OTU 153	ADE/ADJ	AY705967	Basidiomycota	*Agaricomycotina* (no rank)	*Fomitopsis pinicola*
OTU 822	ADE/ADJ	AB111495	Ascomycota	*Pezizomycotina*	*Cordyceps confragosa*
OTU 874	ADE/ADJ	JN054669	nd	nd	Uncultured fungus
OTU 1344	ADE/ADJ	AY584662	Ascomycota	*Pezizomycotina*	*Lithothelium septemseptatum*
OTU 2196	ADE/ADJ	GQ903337	Ascomycota	*Pezizomycotina*	*Aspergillus niger*
OTU 2207	ADE/ADJ	JN941726	Ascomycota	*Pezizomycotina*	*Ophiocordyceps clavata*
OTU 1924	ADE/ADJ	JN054669	nd	nd	Uncultured fungus
OTU 991	ADE	EU688964	Fungi *incertae sedis*	*Mortierellomycotina*	*Mortierellaceae* sp.
OTU 1943	ADE	EU688964	Fungi *incertae sedis*	*Mortierellomycotina*	*Mortierellaceae* sp.
OTU 2141	ADE	EU688964	Fungi *incertae sedis*	*Mortierellomycotina*	*Mortierellaceae* sp.
OTU 2425	ADE	HQ871881	Ascomycota	*Pezizomycotina*	*Plectosphaerella* sp.
OTU 1462	ADE	GQ995336	Chytridiomycota	nd	uncultured Chytridiomycota
OTU 1134	ADE	EF024156	Basidiomycota	*Agaricomycotina* (no rank)	uncultured Boletaceae
OTU 310^b^	ADE	GU369995	nd	nd	Uncultured marine eukaryote
OTU 339^b^	ADE	DQ198797	Basidiomycota	*Pucciniomycotina*	*Atractiella solani*
OTU 362^b^	ADE	GU568155	nd	nd	Uncultured soil fungus
OTU 548^b^	ADE	JN941713	Ascomycota	*Pezizomycotina*	*Ophiocordyceps nutans*
OTU 1526^b^	ADE	AF026592	Basidiomycota	*Agaricomycotina* (no rank)	*Bjerkandera adusta*
OTU 1878^b^	ADE	ABIS01004081	Ascomycota	*Pezizomycotina*	*Coccidioides posadasii*
OTU 2075^b^	ADE	EU417636	Glomeromycota	*Incertae sedis*	Uncultured Glomus
OTU 2282^b^	ADE	AB196322	Fungi *incertae sedis*	*Kickxellomycotina*	*Ramicandelaber longisporus*
OTU 2475^b^	ADE	AB901634	nd	nd	Uncultured eukaryote
OTU118	ADJ	DQ823107	Ascomycota	*Pezizomycotina*	*Exophiala dermatitidis*
OTU 468	ADJ	HQ232212	Ascomycota	*Pezizomycotina*	*Acremonium vitellinum*
OTU 938	ADJ	AF104356	Ascomycota	*Pezizomycotina*	*Pestalosphaeria* sp.
OTU 1002	ADJ	DQ437076	Basidiomycota	*Agaricomycotina* (no rank)	*Cryptococcus aureus*
OTU 1523	ADJ	EF441962	Basidiomycota	nd	Uncultured Basidiomycota
OTU 2120	ADJ	Z30239	Ascomycota	*Pezizomycotina*	*Spathularia flavida*
OTU 2315	ADJ	JF414214	Fungi *incertae sedis*	*Mucoromycotina*	*Mucoromycotina* sp.
OTU 2057	ADJ	JF414228	Fungi *incertae sedis*	*Mucoromycotina*	*Mucoromycotina* sp.
OTU 1853^b^	ADJ	HQ333479	Ascomycota	Mitosporic (no rank)	*Heliocephala gracilis*
OTU 1086^b^	ADJ	AB032629	Basidiomycota	*Agaricomycotina* (no rank)	*Cryptococcus flavus*
OTU 2235^b^	ADJ	JF836023	Ascomycota	*Taphrinomycotina*	*Archaeorhizomyces borealis*
OTU 2242^b^	ADJ	GQ995264	Chytridiomycota	nd	Uncultured Chytridiomycota

*nd* not determined (nd)

^a^Taxonomic classification based on best BLAST hit access after search against SILVA database (97 SILVA 111 taxa map euks) (Quast et al. 2013)

^b^Present only in Balbina, Barro Branco, and Hatahara sites

We also determined the fungal cores in the ADE and ADJ that were present in all samples of the same group but not necessary completely absent in the other one. Due to the particular grouping patterns of the ACU soil samples in the OTU network and CCA analyses (Fig. 1a, b), we decided to compute the fungal core in two ways: including and excluding ACU samples. By considering only the most homogeneous ADE and ADJ sites (BAL, BBO, and HAT), we increased the number of OTUs in the core. The ADE core considering all sites was composed of six OTUs; of those, three had similarity to *Mortierellaceae* sp. (OTUs 991, 1943, and 2141), one to *Plectosphaerella* sp. (OTU 2425), and the other two had similarity to uncultured Chytridiomycota (OTU 1462) and uncultured *Boletaceae* (OTU 1134) (Table 4). After ACU ADE sequence exclusion, the ADE core was increased to 15 OTUs and a higher diversity was observed (Table 4). The ADJ core considering all sites was composed of eight OTUs: two similar to *Mucoromycotina* sp. (OTUs 2315 and 2057) and the others similar to *Exophiala dermatitidis* (OTU 118), *Acremonium vitellinum* (OTU 468), *Pestalosphaeria* sp. (OTU 938), *Cryptococcus aureus* (OTU 1002), uncultured Basidiomycota (OTU 1523), and *Spathularia flavida* (OTU 2120). After ACU ADJ sequence removal, the number of OTUs belonging to ADJ fungal core was raised to 12 OTUs (Table 4).

#### Abundance-Based Analyses

**Fig. 2:**
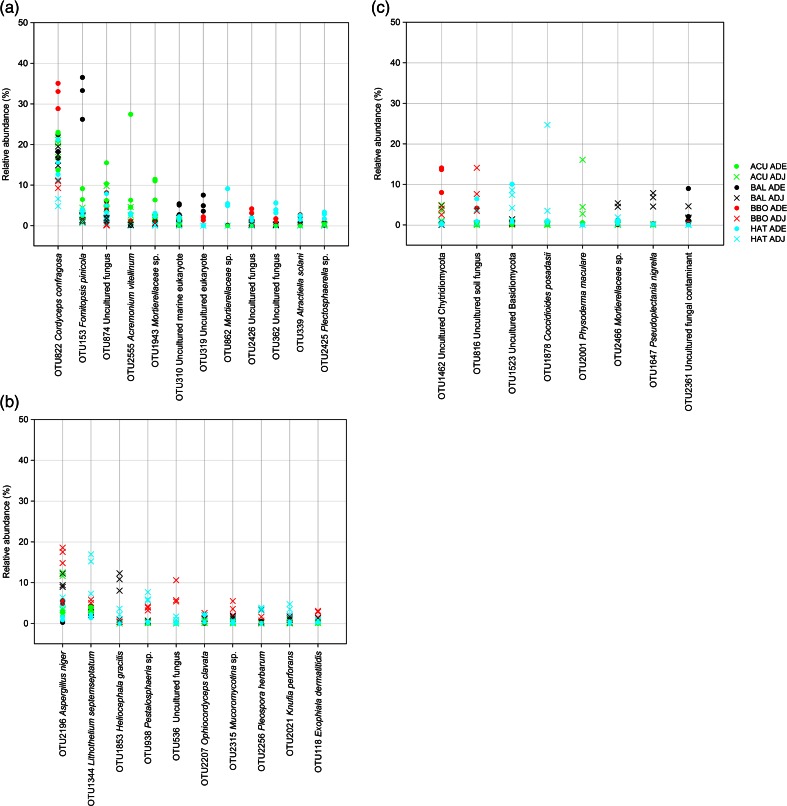
Differential frequencies of most abundant OTUs (>1 % of the group sequences) determined by non-parametric *t* test using Monte Carlo simulation (100 replicates). Plots representing the OTUs statistically significant most abundant in Amazonian Dark Earth (ADE) (**a**), in adjacent (ADJ) soils (**b**), and showing no significant differences between the soil types (**c**)

The dominant OTUs present in at least 75 % of the ADE and ADJ 18S rRNA soil libraries (>1 % of sequences of each group) were identified and tested for statistical significant differences in abundance (non-parametric *t* test followed by Monte Carlo test). Of the 30 OTUs that fit this criterion, 12 were more abundant in ADE soils, 10 in ADJ soils, and 8 showed no significant abundance differences between soil origins (Fig. 2). OTU 822, similar to *C. confragosa*, showed the highest number of 18S rRNA sequences (17.8 %) and was more abundant in the ADE soils (Fig. 2a) than in ADJ soils. The second most abundant was OTU 2196 (6.4 %), similar to *A. niger*, and significantly more abundant in ADJ soils (Fig. 2b) than ADE soils. Chytridiomycota-like OTUs also showed high abundance levels but without differences based on the soil origin (Fig. 2c).

## Discussion

Up to date, the fungal community in ADE has been characterized only by culture-dependent methods [16] and poorly described when compared to Bacteria and Archaea communities [7–10, 12, 31, 32]. To our knowledge, this is the first study assessing the soil fungal composition and diversity of ADE sites in the Brazilian Central Amazonia compared to their respective adjacent non-anthropogenic origin soils by using high-throughput 18S rRNA gene sequencing. No difference in the fungus species richness was observed between ADE and ADJ soils, with the exception of the HAT site, in which higher species richness in ADE was found with the ACE index. This finding diverges from the bacterial community richness that was described being 25 % greater in ADE soils than in ADJ [7]. Culture-dependent [8] and culture-independent analysis [12] also showed a higher bacterial diversity in ADE in comparison with ADJ soils. However, for fungi, we have detected no differences in fungal diversity in ACU and HAT soils. Only the reciprocal of Simpson’s index estimated a lower diversity in BAL and BBO ADE, indicating possible fungal species dominance. Despite the lower fungal species richness and diversity observed in our study, elevated ratios of amino sugar and muramic acid in soil microbial biomass indicated a general predominance of fungi over bacteria in the ADE samples [6].

Previous studies revealed that ADE samples from different origins harbor similar bacterial and archaeal communities as well as bacterial functional genes (e.g., *bph*, encoding for a biphenyl dioxygenase) that are distinct from adjacent soils of non-anthropogenic origin [9, 33]. In this study, we observed the same pattern for fungal communities. The ADE fungal communities in three of the four evaluated sites (BAL, BBO, and HAT) were more similar to each other than their respective ADJ soils at OTU level analysis, thus suggesting an effect of past land use on the fungal community selection. Nevertheless, the same grouping pattern was not observed for the ACU site where the fungal communities could not be segregated by soil type and were more dissimilar from the other ADE and ADJ sites. Currently, the ACU ADE have intensively been used for agriculture under annual crop rotation system (e.g., eggplant, cowpea, cabbage, zucchini, cucumber, passion fruit, papaya) [34] and also showed the lowest amount of organic matter among the surveyed ADE soils. Shifts in fungal communities in Amazonian soils due to land use changes, e.g., conversion of native forest to pasture and agriculture, have already been described [35], but the extension of the alterations in ADE land use on the microbial communities are still scarce [4]. We observed an increase in the common ADE OTUs (ADE fungal core) after ACU sample removal, but we cannot affirm that this effect was a result of the ACU ADE transformations in response to more intensive land use or due to natural differences in ADE ages or formation processes. The low concentration of Al and aluminum saturation in ADJ soil from ACU points to prior lime application before sampling, which could explain the out-grouping of ADJ samples. Lehmann [36] suggests that the specific microbial composition in ADE is a result of its unique conditions rather than the cause. Indeed, the higher amounts of nutrients, mainly P, Ca, Zn, and Mg, and higher SB and V% were associated with ADE fungal communities, whereas Al and aluminum saturations were more associated to the fungal communities in ADJ soils. High levels of Al and Mn indirectly caused by soil pH acidity have been described as a limiting factor for crop production in Amazonian soils [37]. Significant correlations of Al contents in ADE and ADJ soils with bacterial rizhospheric and *bph* gene community structures were also observed [4, 33]; however, further studies are still necessary to confirm this assumptions for fungal communities in these environments.

The microbial functions in ADE are still unclear [36], and most hypothesis relies on black carbon (BC) oxidation, mainly by fungi [6] or BC biological production [38]. Due to its polycyclic aromatic structure, BC cannot be considered an available source of C for microbial growth [3, 39]; however, it may be mineralized by microbial co-metabolism [9]. In our survey, we observed a significantly higher abundance in ADE of OTUs showing similarity to the brown rot fungi *F. pinicola* [40] as well as the saprophytic fungi *A. vitellinum* [41] and *Mortierellaceae* sp. LN07-7-4. A remarkable characteristic of the Basidiomycota brown rot fungi is the selective degradation of wood polysaccharides, which avoids lignin molecules [42]. In the same way, *Mortierella* spp. and *Acremonium* spp. were found in thermophilic compost and vermicompost [43–45]. *Mortierellales* fungi were more associated to manure silage and hay compost than hardwood composts [45]. *Mortierellacea* was also described as dominant in soil samples from primary florets and agricultural areas in Amazonia [35]. Based on these results, we hypothesize that the most abundant fungal species in ADE are involved in the decomposition of fresh organic matter instead of direct oxidation of recalcitrant BC. However, the potential for lower BC oxidation rates by the *Agaricomycotina* fungi cannot be discarded and should be investigated in the future. We were unable to affirm in this study if the decomposition of fresh organic matter priming affected recalcitrant BC decomposition. Controversial results are observed in the literature showing positive effect of glucose on BC oxidation in BC/sandy mixture [46] and no priming effect on BC mineralization by the incorporation of ^13^C-labeled plant residues to ADE in long-term experiments [39]. In the other direction, Glaser and Knorr [38] determined significant amounts of biological BC production under humid tropical conditions and attributed it to the black pigment aspergilin produced by *Aspergillus niger*. Despite the presence of *A. niger* in the general fungal core, the OTU 2196 similar to this species was significantly abundant in the ADJ soils. *A. niger* is a versatile ubiquitous fungus, commonly found in soil and litter [47], and able to produce and secrete enzymes and siderophore molecules [48] and solubilize inorganic P [49].

In this study, we also observed a high abundance of 18S rRNA sequences similar to the fungal species *C. confragosa*, a pathogen of arthropods and other fungal species [50]. Entomopathogenic fungi, like *Cordyceps* and *Ophiocordyceps*, are commonly found in undisturbed tropical humid forests soil and litter and can control insect outbreaks [51]. *C. confragosa*, also known as *Lecanicillium lecanii* (Zimm.) during its anamorphic stage, is a parasite of the green coffee scale (*Coccus viridis*, Hemiptera) [52] and coffee leaf rust fungus (*Hemileia vastatrix*) [53]. In agricultural environments, soil can act as the fungus propagule reservoir during the dry seasons and absence of the target insects [54]. We observed a dominance of *C. confragosa*-like OTUs in the BBO ADE soil that was cultivated with a citrus orchard and speculate that this fungus could be acting in the insect biological control. Further studies are necessary to explore these predictions. Our findings indicated that beyond the importance in C transformations, ADE soils could be a source of new entomopathogenic fungi.

## Conclusions

Our study revealed that fungi communities in ADE were more similar to each other than to the adjacent soils, even when considering the different origins and ages of formation. The concentrations of soil P and Al were the main chemical properties associated to the fungal assemblages in ADE and ADJ soils, respectively. However, other potential factors driving ADE fungi communities beyond the soil chemical attributes might be further investigated. Recently, it was demonstrated that plant species can influence rhizospheric bacterial communities in ADE [4]. The most abundant OTUs in the ADE soils showed similarity to saprophytic fungi species related to fresh organic matter degradation. Studies of the functional diversity of fungi in ADE and the relation with soil organic matter degradation are necessary [31, 36] and should be considered as next step in ADE research.

## Electronic supplementary material

Below is the link to the electronic supplementary material.

ESM 1 (DOCX 579 kb)

## References

[1] Sombroek WG (1966). Amazon soils: a reconnaissence of the soils of the Brazilian Amazon region.

[2] Glaser B (2007). Prehistorically modified soils of central Amazonia: a model for sustainable agriculture in the twenty-first century. Philos Trans R Soc Lond B Biol Sci.

[3] Glaser B, Haumaier L, Guggenberger G, Zech W (2001). The “Terra Preta” phenomenon: a model for sustainable agriculture in the humid tropics. Naturwissenschaften.

[4] Lima AB, Cannavan FS, Navarrete AA (2015). Amazonian Dark Earth and plant species from the Amazon region contribute to shape rhizosphere bacterial communities. Microb Ecol.

[5] Neves EG, Petersen JB, Bartone RN, da Silva CA, Lehmann J (2003). Historical and socio-cultural origins of Amazonian Dark Earths. Amazonian Dark Earths: origin, properties, management.

[6] Glaser B, Birk JJ (2012). State of the scientific knowledge on properties and genesis of anthropogenic Dark Earths in central Amazonia (Terra Preta de Índio). Geochim Cosmochim Acta.

[7] Kim J-S, Sparovek G, Longo RM (2007). Bacterial diversity of Terra Preta and pristine forest soil from the Western Amazon. Soil Biol Biochem.

[8] O’Neill B, Grossman J, Tsai SM (2009). Bacterial community composition in Brazilian Anthrosols and adjacent soils characterized using culturing and molecular identification. Microb Ecol.

[9] Grossman JM, O’Neill BE, Tsai SM (2010). Amazonian Anthrosols support similar microbial communities that differ distinctly from those extant in adjacent, unmodified soils of the same mineralogy. Microb Ecol.

[10] Navarrete AA, Cannavan FS, Taketani RG, Tsai SM (2010). A molecular survey of the diversity of microbial communities in different amazonian agricultural model systems. Diversity.

[11] Roesch LFW, Fulthorpe RR, Riva A (2007). Pyrosequencing enumerates and contrasts soil microbial diversity. ISME J.

[12] Taketani RG, Lima AB, da Conceição Jesus E (2013). Bacterial community composition of anthropogenic biochar and Amazonian Anthrosols assessed by 16S rRNA gene 454 pyrosequencing. Antonie Van Leeuwenhoek.

[13] Kuramae EE, Hillekens RHE, de Hollander M (2013). Structural and functional variation in soil fungal communities associated with litter bags containing maize leaf. FEMS Microbiol Ecol.

[14] Kuramae EE, Verbruggen E, Hillekens R (2013). Tracking fungal community responses to maize plants by DNA- and RNA-based pyrosequencing. PLoS One.

[15] Van der Wal A, Geydan TD, Kuyper TW, de Boer W (2013). A thready affair: linking fungal diversity and community dynamics to terrestrial decomposition processes. FEMS Microbiol Rev.

[16] Ruivo M de LP, Amarante CB do, Oliveira M de LS, Muniz ICM, Santos DAM dos (2009) Microbial population and biodiversity in Amazonian Dark Earth soils. In: Woods WI et al. (eds) Amazonian Dark Earths: Wim Sombroek’s vision. Springer Science, Dordrecht, pp 351–362

[17] Nakamura F, Germano M, Tsai SM (2014). Capacity of aromatic compound degradation by bacteria from Amazon Dark Earth. Diversity.

[18] Rebellato L, Woods WI, Neves EG (2009) Pre-Columbian settlement dynamics in the Central Amazon. In: Woods WI et al. (eds) Amazonian Dark Earths: Wim Sombroek’s vision. Springer Science, Dordrecht, pp 15–32

[19] van Raij B, Andrade JC, Cantarella H, Quaggio JA (2001). Análise química para avaliação de fertilidade de solos tropicais.

[20] Vainio EJ, Hantula J (2000). Direct analysis of wood-inhabiting fungi using denaturing gradient gel electrophoresis of amplified ribosomal DNA. Mycol Res.

[21] Verbruggen E, Kuramae EE, Hillekens R (2012). Testing potential effects of maize expressing the *Bacillus thuringiensis* Cry1AB endotoxin (Bt maize) on mycorrhizal fungal communities via DNA- and RNA-based pyrosequencing and molecular fingerprinting. Appl Environ Microbiol.

[22] Caporaso JG, Kuczynski J, Stombaugh J (2010). QIIME allows analysis of high-throughput community sequencing data. Nat Methods.

[23] Reeder J, Knight R (2010). Rapidly denoising of pyrosequencing amplicon data: exploiting the rank-abundance distribution. Nat Methods.

[24] Edgar RC, Haas BJ, Clemente JC (2011). UCHIME improves sensitivity and speed of chimera detection. Bioinformatics.

[25] Edgar RC (2010). Search and clustering orders of magnitude faster than BLAST. Bioinformatics.

[26] Altschul SF, Gish W, Miller W, Myers EW, Lipman DJ (1990). Basic local alignment search tool. J Mol Biol.

[27] Quast C, Pruesse E, Yilmaz P (2013). The SILVA ribosomal RNA gene database project: improved data processing and web-based tools. Nucleic Acids Res.

[28] Chao A, Wang YT, Jost L (2013). Entropy and the species accumulation curve: a novel entropy estimator via discovery rates of new species. Methods Ecol Evol.

[29] Saito R, Smoot ME, Ono K (2012). A travel guide to cytoscape plugins. Nat Methods.

[30] Hammer Ø, Harper DAT, Ryan PD (2001). Paleontological statistics software package for education and data analysis. Palaeontol Electron.

[31] Tsai SM, O’Neill B, Cannavan FS, Woods WI (2009). The microbial world of Terra Preta. Amazonian Dark Earths: Wim Sombroek’s vision.

[32] Taketani RG, Tsai SM (2010). The influence of different land uses on the structure of archaeal communities in Amazonian Anthrosols based on 16S rRNA and *amo*A genes. Microb Ecol.

[33] Brossi MJDL, Mendes LW, Germano MG (2014). Assessment of bacterial *bph* gene in Amazonian Dark Earth and their adjacent soils. PLoS One.

[34] Falcão NPDS, Borges LF (2006). Efeito da fertilidade de Terra Preta de Índio da Amazônia Central no estado nutricional e na produtividade do mamão hawaí (*Carica papaya* L.). Acta Amaz.

[35] Fracetto GGM, Azevedo LCB, Fracetto FJC (2013). Impact of Amazon land use on the community of soil fungi. Sci Agric.

[36] Lehmann J (2009) Terra Preta Nova—where to from here? In: Woods WI et al. (eds) Amazonian Dark Earths: Wim Sombroek’s vision. Springer Science, Dordrecht, pp 473–486

[37] Falcão NPS, Clemente CR, Tsai SM et al (2009) Pedology, fertility, and biology of Central Amazonian Dark Earths. In: Woods WI et al. (eds) Amazonian Dark Earths: Wim Sombroek’s vision. Springer Science, Dordrecht, pp 213–238

[38] Glaser B, Knorr KH (2008). Isotopic evidence for condensed aromatics from non-pyrogenic sources in soils—implications for current methods for quantifying soil black carbon. Rapid Commun Mass Spectrom.

[39] Liang B, Lehmann J, Sohi SP (2010). Black carbon affects the cycling of non-black carbon in soil. Org Geochem.

[40] Karsten PA (1881). Symbolae ad mycologiam fennicam. Medd af Ocietas pro Fauna Flora Fenn.

[41] Summerbell RC, Gueidan C, Schroers HJ (2011). *Acremonium* phylogenetic overview and revision of *Gliomastix*, *Sarocladium*, and *Trichothecium*. Stud Mycol.

[42] Rabinovich ML, Bolobova AV, Vasil’chenko LG (2004). Fungal decomposition of natural aromatic structures and xenobiotics: a review. Appl Biochem Microbiol.

[43] Anastasi A, Varese GC, Marchisio VF (2005). Isolation and identification of fungal communities in compost and vermicompost. Mycologia.

[44] De Gannes V, Eudoxie G, Hickey WJ (2013). Insights into fungal communities in composts revealed by 454-pyrosequencing: implications for human health and safety. Front Microbiol.

[45] Neher DA, Weicht TR, Bates ST (2013). Changes in bacterial and fungal communities across compost recipes, preparation methods, and composting times. PLoS One.

[46] Hamer U, Marschner B, Brodowski S, Amelung W (2004). Interactive priming of black carbon and glucose mineralisation. Org Geochem.

[47] Klich MA (2002). Biogeography of *Aspergillus* species in soil and litter. Mycologia.

[48] Pel HJ, de Winde JH, Archer DB (2007). Genome sequencing and analysis of the versatile cell factory *Aspergillus niger* CBS 513.88. Nat Biotechnol.

[49] Chuang CC, Kuo YL, Chao CC, Chao WL (2007). Solubilization of inorganic phosphates and plant growth promotion by *Aspergillus niger*. Biol Fertil Soils.

[50] Sung GH, Hywel-Jones NL, Sung JM (2007). Phylogenetic classification of *Cordyceps* and the clavicipitaceous fungi. Stud Mycol.

[51] Augustyniuk-Kram A, Kram KJ, Blanco AJ (2012). Entomopathogenic fungi as an important natural regulator of insect outbreaks in forests. Forest ecossistems - more than just trees.

[52] Viégas AP (1939). Um amigo do fazendeiro Verticillium lecanii (Zimm.).n comb. O causador do halo branco do *Coccus viridis* Green. Rev do Inst do Café do Estado São Paulo.

[53] Jackson D, Skillman J, Vandermeer J (2012). Indirect biological control of the coffee leaf rust, *Hemileia vastatrix*, by the entomogenous fungus *Lecanicillium lecanii* in a complex coffee agroecosystem. Biol Control.

[54] Jackson D, Zemenick K, Huerta G (2012). Occurrence in the soil and dispersal of *Lecanicillium lecanii*, a fungal pathogen of the green coffee scale (*Coccus viridis*) and coffee rust (*Hemileia vastatrix*). Trop Subtrop Agroecosyst.

