# Supplementary material for: Fungal Community Assembly in the Amazonian Dark Earth
Source: Microb Ecol. 2015 Nov 19;71:962–73. doi: 10.1007/s00248-015-0703-7 (PMC4823338; doi:10.1007/s00248-015-0703-7)
Supplement: Supplementary file 1 — (DOCX 579 kb) [file 248_2015_703_MOESM1_ESM.docx]

**Fungal community assembly in the Amazonian Dark Earth**

Adriano Reis Lucheta^1^**^†^**, Fabiana de Souza Cannavan^2^**^†^**, Luiz Fernando Wurdig Roesch^3^, Siu Mui Tsai^2^, Eiko Eurya Kuramae^1*^

^1^Department of Microbial Ecology, Netherlands Institute of Ecology (NIOO/KNAW), Wageningen, The Netherlands

^2^Centro de Energia Nuclear na Agricultura (CENA), Universidade de São Paulo (USP), Piracicaba, Brasil

^3^Centro Interdisciplinar de Pesquisas em Biotecnologia (CIP-Biotec), Universidade Federal do Pampa, São Gabriel, Brasil

† These authors share first authorship.

*Corresponding Author: Eiko E. Kuramae, Department of Microbial Ecology, Netherlands Institute of Ecology (NIOO-KNAW), Droevendaalsesteeg 10, Wageningen 6708 PB, The Netherlands. Tel.: +31(0)317 47 35 02; e-mail: e.kuramae@nioo.knaw.nl

**Online Resource 1** – Schematic design used for soil sampling. Samples selected for DNA extraction were represented in yellow.

**
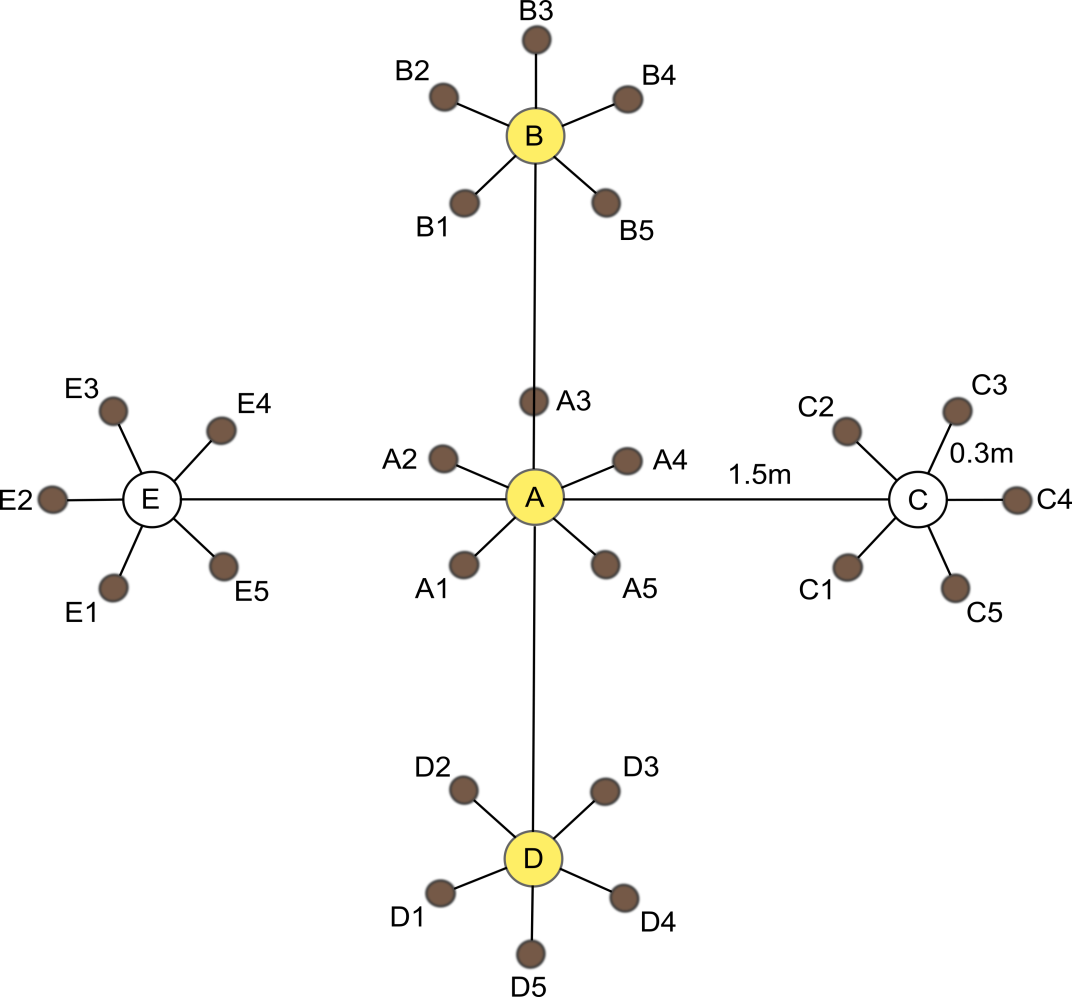
**

**Online Resource 2 -** List of barcoded primers used for 18S rRNA gene amplification and 454 pyrosequencing of Açutuba (ACU), Balbina (BAL), Barro Branco (BBO) and Hatahara (HAT) Amazonian Dark Earth (ADE) and adjacent (ADJ) soil samples.

| Site | Soil Type | Replicate | Primer Name | Primer Sequence | Key | MID |
| --- | --- | --- | --- | --- | --- | --- |
| ACU | ADE | 1 | FR1(MID-67) | AICCATTCAATCGGTAIT | CGTATCGCCTCCCTCGCGCCATCAG | TCGATAGTGA |
|  |  |  | FF390w(MID-67) | CGWTAACGAACGAGACCT | CTATGCGCCTTGCCAGCCCGCTCAG | TCGATAGTGA |
| ACU | ADE | 2 | FR1(MID-68) | AICCATTCAATCGGTAIT | CGTATCGCCTCCCTCGCGCCATCAG | TCGCTGCGTA |
|  |  |  | FF390w(MID-68) | CGWTAACGAACGAGACCT | CTATGCGCCTTGCCAGCCCGCTCAG | TCGCTGCGTA |
| ACU | ADE | 3 | FR1(MID-69) | AICCATTCAATCGGTAIT | CGTATCGCCTCCCTCGCGCCATCAG | TCTGACGTCA |
|  |  |  | FF390w(MID-69) | CGWTAACGAACGAGACCT | CTATGCGCCTTGCCAGCCCGCTCAG | TCTGACGTCA |
| ACU | ADJ | 1 | FR1(MID-67) | AICCATTCAATCGGTAIT | CGTATCGCCTCCCTCGCGCCATCAG | TCGATAGTGA |
|  |  |  | FF390w(MID-67) | CGWTAACGAACGAGACCT | CTATGCGCCTTGCCAGCCCGCTCAG | TCGATAGTGA |
| ACU | ADJ | 2 | FR1(MID-68) | AICCATTCAATCGGTAIT | CGTATCGCCTCCCTCGCGCCATCAG | TCGCTGCGTA |
|  |  |  | FF390w(MID-68) | CGWTAACGAACGAGACCT | CTATGCGCCTTGCCAGCCCGCTCAG | TCGCTGCGTA |
| ACU | ADJ | 3 | FR1(MID-69) | AICCATTCAATCGGTAIT | CGTATCGCCTCCCTCGCGCCATCAG | TCTGACGTCA |
|  |  |  | FF390w(MID-69) | CGWTAACGAACGAGACCT | CTATGCGCCTTGCCAGCCCGCTCAG | TCTGACGTCA |
| BAL | ADE | 1 | FR1(MID-24) | AICCATTCAATCGGTAIT | CGTATCGCCTCCCTCGCGCCATCAG | TAGAGACGAG |
|  |  |  | FF390w(MID-24) | CGWTAACGAACGAGACCT | CTATGCGCCTTGCCAGCCCGCTCAG | TAGAGACGAG |
| BAL | ADE | 2 | FR1(MID-25) | AICCATTCAATCGGTAIT | CGTATCGCCTCCCTCGCGCCATCAG | TCGTCGCTCG |
|  |  |  | FF390w(MID-25) | CGWTAACGAACGAGACCT | CTATGCGCCTTGCCAGCCCGCTCAG | TCGTCGCTCG |
| BAL | ADE | 3 | FR1(MID-26) | AICCATTCAATCGGTAIT | CGTATCGCCTCCCTCGCGCCATCAG | ACATACGCGT |
|  |  |  | FF390w(MID-26) | CGWTAACGAACGAGACCT | CTATGCGCCTTGCCAGCCCGCTCAG | ACATACGCGT |
| BAL | ADJ | 1 | FR1(MID-24) | AICCATTCAATCGGTAIT | CGTATCGCCTCCCTCGCGCCATCAG | TAGAGACGAG |
|  |  |  | FF390w(MID-24) | CGWTAACGAACGAGACCT | CTATGCGCCTTGCCAGCCCGCTCAG | TAGAGACGAG |
| BAL | ADJ | 2 | FR1(MID-25) | AICCATTCAATCGGTAIT | CGTATCGCCTCCCTCGCGCCATCAG | TCGTCGCTCG |
|  |  |  | FF390w(MID-25) | CGWTAACGAACGAGACCT | CTATGCGCCTTGCCAGCCCGCTCAG | TCGTCGCTCG |
| BAL | ADJ | 3 | FR1(MID-26) | AICCATTCAATCGGTAIT | CGTATCGCCTCCCTCGCGCCATCAG | ACATACGCGT |
|  |  |  | FF390w(MID-26) | CGWTAACGAACGAGACCT | CTATGCGCCTTGCCAGCCCGCTCAG | ACATACGCGT |
| BBO | ADE | 1 | FR1(MID-61) | AICCATTCAATCGGTAIT | CGTATCGCCTCCCTCGCGCCATCAG | CTATAGCGTA |
|  |  |  | FF390w(MID-61) | CGWTAACGAACGAGACCT | CTATGCGCCTTGCCAGCCCGCTCAG | CTATAGCGTA |
| BBO | ADE | 2 | FR1(MID-62) | AICCATTCAATCGGTAIT | CGTATCGCCTCCCTCGCGCCATCAG | TACGTCATCA |
|  |  |  | FF390w(MID-62) | CGWTAACGAACGAGACCT | CTATGCGCCTTGCCAGCCCGCTCAG | TACGTCATCA |
| BBO | ADE | 3 | FR1(MID-63) | AICCATTCAATCGGTAIT | CGTATCGCCTCCCTCGCGCCATCAG | TAGTCGCATA |
|  |  |  | FF390w(MID-63) | CGWTAACGAACGAGACCT | CTATGCGCCTTGCCAGCCCGCTCAG | TAGTCGCATA |
| BBO | ADJ | 1 | FR1(MID-61) | AICCATTCAATCGGTAIT | CGTATCGCCTCCCTCGCGCCATCAG | CTATAGCGTA |
|  |  |  | FF390w(MID-61) | CGWTAACGAACGAGACCT | CTATGCGCCTTGCCAGCCCGCTCAG | CTATAGCGTA |
| BBO | ADJ | 2 | FR1(MID-62) | AICCATTCAATCGGTAIT | CGTATCGCCTCCCTCGCGCCATCAG | TACGTCATCA |
|  |  |  | FF390w(MID-62) | CGWTAACGAACGAGACCT | CTATGCGCCTTGCCAGCCCGCTCAG | TACGTCATCA |
| BBO | ADJ | 3 | FR1(MID-63) | AICCATTCAATCGGTAIT | CGTATCGCCTCCCTCGCGCCATCAG | TAGTCGCATA |
|  |  |  | FF390w(MID-63) | CGWTAACGAACGAGACCT | CTATGCGCCTTGCCAGCCCGCTCAG | TAGTCGCATA |
| HAT | ADE | 1 | FR1(MID-64) | AICCATTCAATCGGTAIT | CGTATCGCCTCCCTCGCGCCATCAG | TATATATACA |
|  |  |  | FF390w(MID-64) | CGWTAACGAACGAGACCT | CTATGCGCCTTGCCAGCCCGCTCAG | TATATATACA |
| HAT | ADE | 2 | FR1(MID-65) | AICCATTCAATCGGTAIT | CGTATCGCCTCCCTCGCGCCATCAG | TATGCTAGTA |
|  |  |  | FF390w(MID-65) | CGWTAACGAACGAGACCT | CTATGCGCCTTGCCAGCCCGCTCAG | TATGCTAGTA |
| HAT | ADE | 3 | FR1(MID-66) | AICCATTCAATCGGTAIT | CGTATCGCCTCCCTCGCGCCATCAG | TCACGCGAGA |
|  |  |  | FF390w(MID-66) | CGWTAACGAACGAGACCT | CTATGCGCCTTGCCAGCCCGCTCAG | TCACGCGAGA |
| HAT | ADJ | 1 | FR1(MID-64) | AICCATTCAATCGGTAIT | CGTATCGCCTCCCTCGCGCCATCAG | TATATATACA |
|  |  |  | FF390w(MID-64) | CGWTAACGAACGAGACCT | CTATGCGCCTTGCCAGCCCGCTCAG | TATATATACA |
| HAT | ADJ | 2 | FR1(MID-65) | AICCATTCAATCGGTAIT | CGTATCGCCTCCCTCGCGCCATCAG | TATGCTAGTA |
|  |  |  | FF390w(MID-65) | CGWTAACGAACGAGACCT | CTATGCGCCTTGCCAGCCCGCTCAG | TATGCTAGTA |
| HAT | ADJ | 3 | FR1(MID-66) | AICCATTCAATCGGTAIT | CGTATCGCCTCCCTCGCGCCATCAG | TCACGCGAGA |
|  |  |  | FF390w(MID-66) | CGWTAACGAACGAGACCT | CTATGCGCCTTGCCAGCCCGCTCAG | TCACGCGAGA |

**Online Resource 3 –** Summary of the number of sequences and OTUs after each bioinformatics steep.

|  | UCLUST Clustering | Filter by Taxonomy (Fungi Domain only) | Singletons Removal | Rarefaction |
| --- | --- | --- | --- | --- |
| Number of Samples | 24 | 24 | 24 | 24 |
| Number of Total Sequences | 132 764 | 105 579 | 105 019 | 41 472 |
| Min. | 2232 | 1742 | 1728 | 1728 |
| Max. | 8477 | 6734 | 6712 | 1728 |
| Median | 5826 | 4373 | 4355 | 1728 |
| Mean | 5531.8 | 4395 | 4375.8 | 1728 |
| Std. dev. | 1397.1 | 1156.7 | 1155.3 | 0.0 |
| Number of OTUs | 2568 | 1420 | 960 | 859 |
|  | |  |  |  |
| *Number of sequences per sample* | |  |  |  |
| BAL ADE R1 | 4986 | 3865 | 3844 | 1728 |
| BAL ADE R2 | 5210 | 3818 | 3793 | 1728 |
| BAL ADE R3 | 6040 | 4158 | 4139 | 1728 |
| BAL ADJ R1 | 4143 | 3170 | 3150 | 1728 |
| BAL ADJ R2 | 2232 | 1742 | 1728 | 1728 |
| BAL ADJ R3 | 3595 | 2699 | 2688 | 1728 |
| BBO ADE R1 | 5741 | 4697 | 4673 | 1728 |
| BBO ADE R2 | 8111 | 6734 | 6712 | 1728 |
| BBO ADE R3 | 6282 | 5303 | 5277 | 1728 |
| BBO ADJ R1 | 6068 | 4369 | 4352 | 1728 |
| BBO ADJ R2 | 5138 | 3667 | 3648 | 1728 |
| BBO ADJ R3 | 5391 | 3754 | 3739 | 1728 |
| ACU ADE R1 | 5941 | 4902 | 4890 | 1728 |
| ACU ADE R2 | 8477 | 6324 | 6303 | 1728 |
| ACU ADE R3 | 6139 | 4847 | 4829 | 1728 |
| ACU ADJ R1 | 5911 | 4816 | 4793 | 1728 |
| ACU ADJ R2 | 6016 | 4888 | 4873 | 1728 |
| ACU ADJ R3 | 6877 | 5813 | 5789 | 1728 |
| HAT ADE R1 | 5087 | 4053 | 4030 | 1728 |
| HAT ADE R2 | 7142 | 5625 | 5604 | 1728 |
| HAT ADE R3 | 3796 | 3166 | 3138 | 1728 |
| HAT ADJ R1 | 6031 | 5558 | 5545 | 1728 |
| HAT ADJ R2 | 3524 | 3134 | 3124 | 1728 |
| HAT ADJ R3 | 4886 | 4377 | 4358 | 1728 |

**Online Resource 4** – Rarefaction curves representing the number of observed species according to the soil samples sequencing effort (A), or after sampling merge according to sampling location (B) or soil type (C).

**
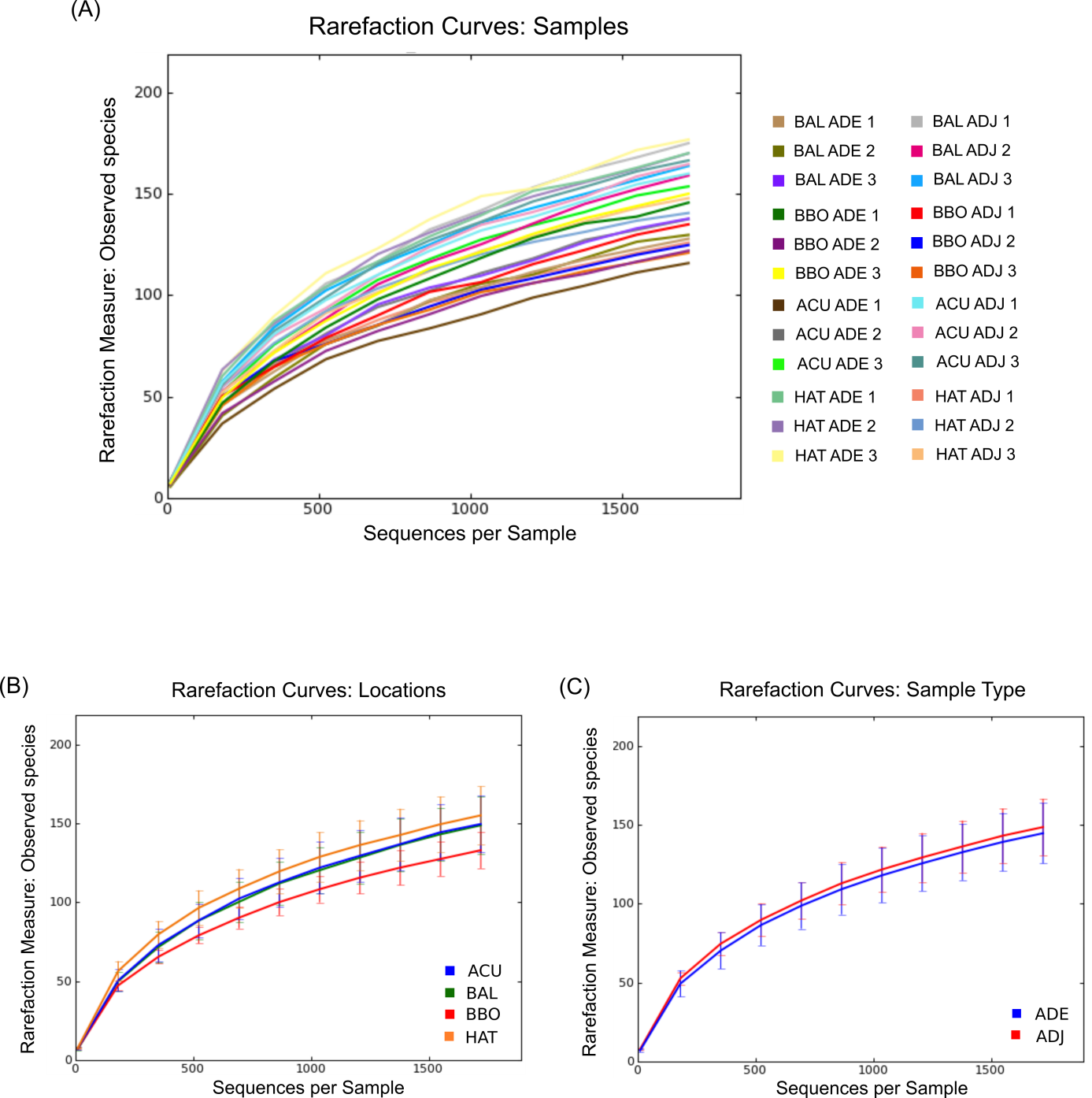
**

**Online Resource 5 -** Contribution of soil physico-chemical and fertility attributes in the dissimilarities of Amazonian Dark Earth (ADE) and adjacent (ADJ) soils fungal communities calculated by similarity percentage analysis (SIMPER) using Bray-Curtis distance.

| Soil Properties | Average Dissimilarities | Contribution (%) | Cumulative (%) |
| --- | --- | --- | --- |
| P | 3.8 | 13.7 | 13.7 |
| m% | 3.7 | 13.3 | 27.0 |
| Ca | 2.9 | 10.3 | 37.4 |
| SB | 2.6 | 9.4 | 46.8 |
| Al | 2.5 | 8.9 | 55.7 |
| Zn | 2.3 | 8.5 | 64.1 |
| V% | 2.2 | 7.9 | 72.1 |
| Mn | 1.7 | 6.3 | 78.3 |
| Mg | 1.4 | 5.0 | 83.4 |
| Fe | 1.3 | 4.7 | 88.1 |
| Cu | 1.0 | 3.4 | 91.5 |
| H+Al | 0.7 | 2.6 | 94.1 |
| OM | 0.7 | 2.5 | 96.6 |
| CEC | 0.5 | 1.8 | 98.4 |
| pH | 0.3 | 0.9 | 99.3 |
| B | 0.2 | 0.7 | 100.0 |

Organic matter (OM); cation exchange capacity in pH 7 (CEC); sum of bases (SB); V- soil base saturation index (%). m- Al saturation index.
